# ARM-Net: A Tri-Phase Integrated Network for Hyperspectral Image Compression

**DOI:** 10.3390/s25061843

**Published:** 2025-03-16

**Authors:** Qizhi Fang, Zixuan Wang, Jingang Wang, Lili Zhang

**Affiliations:** 1 Liaoning General Aviation Academy, Shenyang 110136, China; 2 School of Electronical and Information Engineering, Shenyang Aerospace University, Shenyang 110136, China

**Keywords:** hyperspectral image compression, spectral reconstruction, spatial-spectral attention mechanism, recurrent spectral attention mechanism

## Abstract

Most current hyperspectral image compression methods rely on well-designed modules to capture image structural information and long-range dependencies. However, these modules tend to increase computational complexity exponentially with the number of bands, which limits their performance under constrained resources. To address these challenges, this paper proposes a novel triple-phase hybrid framework for hyperspectral image compression. The first stage utilizes an adaptive band selection technique to sample the raw hyperspectral image, which mitigates the computational burden. The second stage concentrates on high-fidelity compression, efficiently encoding both spatial and spectral information within the sampled band clusters. In the final stage, a reconstruction network compensates for sampling-induced losses to precisely restore the original spectral details. The proposed framework, known as ARM-Net, is evaluated on seven mixed hyperspectral datasets. Compared to state-of-the-art methods, ARM-Net achieves an overall improvement of approximately 1–2 dB in both the peak signal-to-noise ratio and multiscale structural similarity index measure, as well as a reduction in the average spectral angle mapper of approximately 0.1.

## 1. Introduction

Hyperspectral imaging is a broadly adopted data acquisition technique in which hyperspectral sensors mounted on spaceborne or airborne platforms capture narrowband continuous spectral images of the Earth’s surface [1,2]. These sensors can capture a wide spectral range, from visible light to shortwave infrared, with each pixel containing spectral information that is reflected or radiated from multiple bands. Typically, a hyperspectral image (HSI) is represented as a three-dimensional data cube that captures spatial and spectral information about the physical world and characterizes the intrinsic optical properties of each location. Hyperspectral imaging has become increasingly popular in real-world agricultural and industrial monitoring due to its ability to detect subtle variations across numerous spectral bands. In precision agriculture, for example, HSI data enable detailed assessment of crop health and early detection of diseases or nutrient deficiencies. Such insights support better resource allocation and yield optimization while mitigating environmental impacts. In industrial settings, hyperspectral imaging can be employed for quality inspection and material identification, which facilitates faster and more reliable defect detection in manufacturing processes. Moreover, the growing availability of spaceborne or UAV-based hyperspectral sensors has paved the way for large-scale applications in monitoring, surveying, and real-time decision-making across diverse domains. Despite these clear advantages, the fine spectral resolution of HSIs generates large data volumes and exhibits significant spectral redundancy, which not only poses substantial challenges for storage and transmission but also limits its efficient application in downstream tasks [3,4,5,6,7]. As hyperspectral sensors proliferate in agricultural and industrial operations, the exponential growth of data volume compounds these bottlenecks. Hence, the efficient storage and transmission of HSI data have become critical issues that require urgent attention, prompting extensive research into advanced compression techniques and algorithmic innovations.

Transform coding has demonstrated significant effectiveness in HSI compression. A common approach involves applying Principal Component Analysis (PCA) to decorrelate the spectra and reduce the spectral dimensionality of HSIs. Subsequently, the dimensionally reduced data can be compressed in blocks or at multiple resolutions by the Joint Photographic Experts Group (JPEG) [8] or JPEG2000 [9] to further optimize data storage and transmission. However, these methods [8,9] inevitably lose critical spectral information and image details (e.g., textures and edges) at high compression ratios, resulting in significant degradation of image quality and making the compression techniques unsuitable for applications that require high fidelity. Autoencoders or variational autoencoders [10,11] based on rate distortion (RD) [12] have paved the way for new developments in lossy compression methods. In contrast to the aforementioned traditional mathematical methods, convolutional neural network-based codecs have effectively addressed the limitations of traditional algorithms by simultaneously optimizing reconstruction distortion and compression ratio. This architecture has been widely applied to compress RGB images [13,14,15,16,17,18,19], multispectral images [20], and HSIs [21,22,23,24,25,26], with optimizations tailored for each component of the framework.

Deep learning techniques have found extensive application in RGB image compression. These methods aim to optimize the compression rate or reduce reconstructive distortion by tailored modules. Specifically, Minnen [13] jointly combined an autoregressive mask and a hyperprior for accurate probabilistic modeling, but the introduction of serial operations resulted in longer decoding times. To tackle the decoding efficiency issue, He [14] introduced an optimized checkerboard context model, building upon the work of [13], to enable parallel decoding. This approach significantly enhances decoding efficiency by improving parallelization and computational effectiveness. In addition, the combination of a hyperprior and local context results in a limited global perspective. To overcome this limitation, Qian [15] introduced an approach integrating local and global contextual information with a hyperprior, resulting in an enhanced compression rate. However, the global context entropy model exhibits quadratic complexity, which makes it computationally intensive. To tackle this issue, Jiang [16] introduced a multi-reference entropy model with linear complexity, which effectively reduces the computational load. On the other hand, Cheng [17] utilized discretized Gaussian mixture likelihood to model pixel distribution and employed a non-local attention module for global feature extraction. Han [18] achieved efficient remote sensing image compression through a combination of an edge-guiding mechanism and adversarial learning, preserving image edges and texture details. Tang [19] addressed the limitations of traditional deep image compression methods through a combination of graph attention mechanisms and asymmetric convolutional neural networks based on autoregressive modeling.

HSIs contain more spectral information than RGB images, which introduces new challenges for compression tasks. In HSIs, the limited spatial resolution and substantial spectral overlap degrade compression performance. Kong [20] designed a neuroscience-based non-local attention module that captures both fine features of nearby pixels and large-scale features in the spatial domain. Additionally, a multi-scale spectral attention block was introduced to extract non-smooth spectral correlations at different scales. Guo [21] introduced an edge component in RD loss combined with an interactive dual attention module to enhance the integrated structure of the latent representations. Additionally, Guo [22] explored HSI channel-level relationships and used contrastive informative feature encoding to address the problem of collapsing and losing information attributes at high compression ratios. Rezasoltani [23] and Zhang [24] used implicit neural representations and neural radiation fields, respectively, to map HSIs to low-dimensional representations, thus effectively alleviating the compression difficulty. Byju [25] used 3D-Conv and squeeze-and-excitation attention modules jointly to eliminate HSI spatial and spectral redundancies. Sebastià Mijares i Verdú [26] employed a channel clustering strategy to address the computational complexity and scalability issues.

Although the aforementioned methods have made significant progress in HSI compression, there is still potential for improvement in achieving more efficient compression. First, these well-designed modules focus only on specific dimensions (spatial or spectral) and fail to jointly eliminate their redundancies. Second, increasing the number of parameters in these networks is necessary to compress images with larger numbers of bands; that is, their performance cannot be maintained as the number of bands increases [26]. Moreover, well-designed feature extraction modules can lead to an explosion in Floating-Point Operations per Second (FLOPs) when handling high-dimensional inputs, which ultimately degrades network performance. To overcome these challenges, we propose a three-stage framework for hyperspectral compression. The first stage utilizes an adaptive band selector for initial spectral feature extraction on high-dimensional HSIs, followed by the extraction of key spectral features most relevant to subsequent processing. The selected band clusters aim to represent the complete information of HSIs while reducing redundant data, which optimizes the efficiency of the entire compression framework and enables a more efficient representation of HSI data. The second stage utilizes a compression network integrated with a multi-head recurrent spectral attention mechanism to perform secondary compression, which achieves the highest compression ratio. This architecture leverages multiple attention heads to selectively focus on different spectral features across various bands, which enhances the model’s ability to capture intricate relationships within the spectral data. Through a cyclical focus on different portions of the spectrum, the compression network ensures that the most relevant features are identified and prioritized, leading to a maximized compression ratio and minimized information loss. The third stage uses a multi-scale spatial-spectral attention-based reconstruction network for spatial and spectral reconstruction to decompress the original high-dimensional input. The network integrates local and global spatial and spectral information to construct a high-precision reconstruction network, which is refined from coarse to fine and then integrated to obtain a high-fidelity reconstruction of the original HSI.

The main contributions of this work are outlined as follows:

1. This research proposes an innovative three-stage hyperspectral compression framework, known as ARM-Net. ARM-Net consists of an adaptive band selector (ABS), a Recurrent Spectral Attention Compression Network (RSACN), and a Multi-Scale Spatial-Spectral Attention Reconstruction Network (MSSARN).
2. To alleviate the burden on the compression network, this paper introduces the ABS, which builds upon a common band selection mechanism used in hyperspectral lossless compression. By adaptively selecting band clusters with the highest information content, the ABS reduces the overall computational load of the framework.
3. To enhance hyperspectral compression, ARM-Net incorporates a multi-head recurrent spectral attention (MHRSA) module within its codec. MHRSA dynamically assigns attention weights to spectral bands, allowing the network to focus on the most relevant spectral features for compression. By leveraging multiple attention heads, the module captures diverse spectral interactions to preserve spectral consistency across bands, resulting in reduced redundancy and improved compression efficiency. This targeted weight adjustment approach is essential to address varying spectral pixel values, mitigating information loss that simple averaging methods may overlook.
4. To optimize hyperspectral reconstruction, we propose a Spatial-Spectral Attention Block (SSAB) within the reconstruction backbone of ARM-Net. The SSAB jointly models spatial and spectral dependencies to enhance reconstruction accuracy, which compensates for spatial detail loss during compression. Spectral-Wise Multi-Head Self-Attention (Spec-MSA) and Spatial Multi-Head Self-Attention (Spa-MSA) in the SSAB are linked by residuals to effectively compensate for the lack of spatial details in HSI reconstruction through spectral reconstruction (SR) networks. This versatile and efficient plug-and-play spatial-spectral attention mechanism captures fine-grained features across both spatial and spectral dimensions while preserving a linear relationship between spatial dimensions and computational complexity.
5. We comprehensively evaluate the network on our mixed hyperspectral dataset. Experimental results demonstrate that ARM-Net surpasses state-of-the-art (SOTA) approaches in terms of the peak signal-to-noise ratio (PSNR), multi-scale structural similarity index measure (MS-SSIM), and spectral angle mapper (SAM).

## 2. Methods

### 2.1. The Proposed Three-Stage Compression Framework

**Figure 1:**
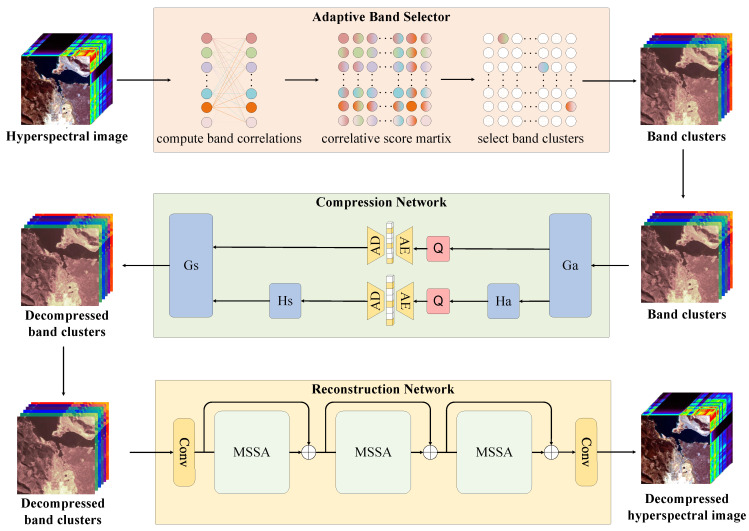
The overall structure of the proposed framework, with ARM-Net’s structure illustrated as the final network.

Figure 1 illustrates the overall structure of the proposed three-stage compression framework and highlights the key components of ARM-Net: the ABS, RSACN, and MSSARN. Let $X_{in}\in R^{H\times W\times C}$ represent the input HSI, where H denotes the height, W is its width, and C is the number of bands. The compression process of our framework for HSIs is as follows: $X_{in}$ is first passed through a band selection network to identify band clusters that effectively represent the entire HSI. Within ARM-Net, the spectral correlation coefficient upper triangular matrix is computed, and β pairs of bands with lower scores are selected as input band clusters $X_{\beta }\in R^{H\times W\times 2\beta }$ for the compression network. The compression network performs secondary compression on $X_{\beta }$ to obtain the bitstream with the maximum compression ratio. Within ARM-Net, the RSACN further enhances the ability to eliminate spectral redundancy, resulting in a higher compression ratio. After the maximally compressed bitstream is obtained, the decoder reconstructs it into sampled cluster bands $\hat{X_{\beta }}$, which are then passed to the reconstruction network. Finally, the reconstruction network reconstructs $\hat{X_{T}}$ into original-dimensional data $\hat{X_{in}}$. The whole compression process of ARM-Net can be expressed by Equations (1)–(3):

(1) $$X_{\beta }=\mathrm{ABS}\left(X_{\mathrm{in}}\right)$$

(2) $$\hat{X_{\beta }}=\mathrm{RSACN}\left(X_{\beta }\right)$$

(3) $${\hat{X}}_{\mathrm{in}}=\mathrm{MSSARN}\left(\hat{X_{\beta }}\right)$$

### 2.2. Adaptive Band Selector (ABS)

In response to the high-dimensional nature of HSIs, the band selection technique effectively reduces computational complexity and optimizes data storage and transmission efficiency. A few representative bands are chosen to reduce equipment costs and computational load while maintaining data expressiveness. Therefore, band selection and extraction are typically regarded as fundamental steps in lossless compression algorithms [27,28] and compressed sensing [29,30].

**Figure 2:**
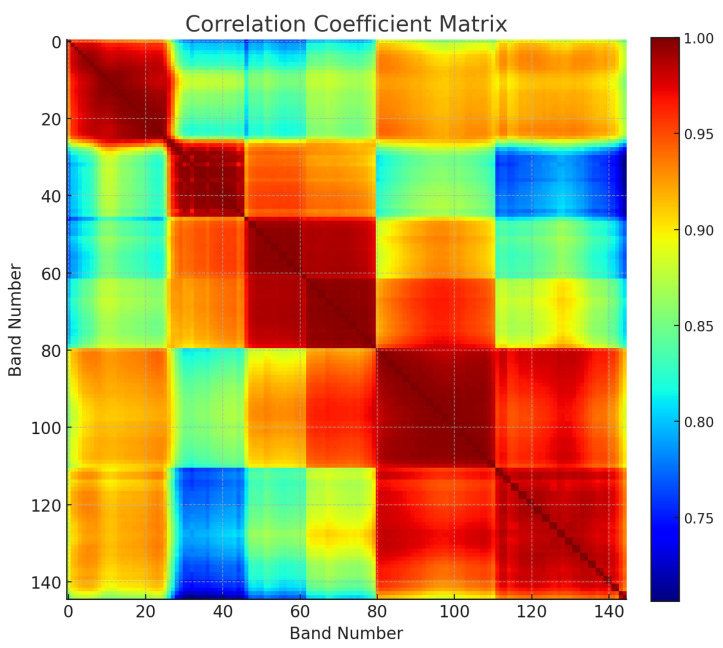
Example of cross-correlation between spectral bands for the Botswana dataset.

Band selection employs metrics such as entropy, interquartile range, standard deviation, and image gradient to flexibly evaluate the relevance of each band and selects bands with high information content, strong variability, or prominent texture features based on different criteria. However, according to the fundamental principles of information theory [31], higher information content in the selected bands does not necessarily result in better performance of the band combination. Instead, inter-band redundancy must be considered to achieve optimal information utilization and overall performance optimization. Thus, in hyperspectral data analysis, maximizing the information capacity of the selected bands while minimizing inter-band redundancy is crucial for optimizing hyperspectral data processing frameworks. In our framework, reconstruction accuracy depends on the amount of information in the sampled band clusters. In other words, if a band cluster fails to fully represent the corresponding HSI patch, the compression process becomes ineffective. To more comprehensively represent the full-band spectral information, this study introduces spectral correlation coefficients based on the maximum correlation principle [32] to quantify the spectral correlation between bands and enhance data independence through spectral decorrelation techniques, as follows:

(4) $$c_{i,j}=\frac{\sum_{x=1}^{H}\sum_{y=1}^{W}\left[p_{i}\left(x,y\right)-{\bar{p}}_{i}\right]\left[p_{j}\left(x,y\right)-{\bar{p}}_{j}\right]}{\sqrt{\sum_{x=1}^{H}\sum_{y=1}^{W}{\left[p_{i}\left(x,y\right)-{\bar{p}}_{i}\right]}^{2}\sum_{x=1}^{H}\sum_{y=1}^{W}{\left[p_{j}\left(x,y\right)-{\bar{p}}_{j}\right]}^{2}}}$$

where *i* and *j* represent the *i*-th and *j*-th bands in the HSI patch, respectively; $p_{i}\left(x,y\right)$ represents the pixel value at position (x,y) of the *i*-th band; and ${\bar{p}}_{i}$ denotes the mean pixel value of the *i*-th band. Figure 2 presents a sample correlation matrix of the Botswana dataset. It can be observed that adjacent bands generally exhibit a high degree of correlation, with many local bands having correlation coefficients greater than 0.95, which indicates significant information redundancy. The pseudo-code in Algorithm 1 provides a detailed description of the adaptive band selection strategy, enabling the adaptive computation of optimal band clusters for new HSIs. In this study, the ABS is applied to cropped HSI patches, and the β pairs of bands that appear most frequently across all results are selected as the final adaptive selection outcome. Based on experimental results, the hyperparameter β is set to 3, and the band cluster [1, 2, 20, 25, 29, 30] is selected for training. Detailed experimental procedures and analyses are provided in the ablation experiments section, where the effectiveness of the selected band clusters is validated.
**Algorithm 1** Adaptive band selection algorithm workflow**Input:** HSI $X_{in}\in R^{H\times W\times C}$**Output:** Selected band pairs *L*Initialize an empty list L to store selected band pairs**for** i = 1 **to** C − 1 **do**   **for** j = i + 1 **to** C **do**      calculate inter-spectral correlation coefficients $C_{i,j}$   **end for****end for****for** k=1 **to** β **do**   Select the *k*-th pair of bands (i,j) with the lowest correlation from the sorted list   **if** band *i* or *j* has already been selected **then**      Skip the current pair and move to the next pair in the sorted list   **else**      Append (i,j) to the list L   **end if****end for****if** less than β pairs are selected **then**   Continue searching for additional band pairs to complete the selection if necessary**end if**returns the list of selected band pairs L

### 2.3. Recurrent Spectral Attention Compression Network (RSACN)

The compression network is crucial for enhancing the compression ratio and plays a pivotal role in the three-stage framework. This section first presents the overall architecture of the RSACN. Next, a detailed explanation of the multi-head recurrent spectral attention (MRSA) module is provided, highlighting its design and improvements.

**Figure 3:**
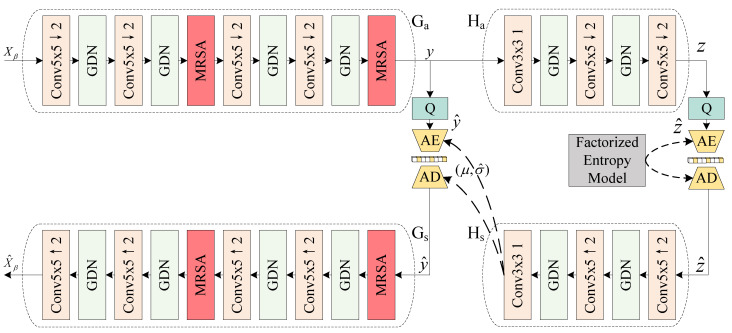
The backbone of the Recurrent Spectral Attention Compression Network (RSACN) based on a variational autoencoder. The red MRSA is our multi-head recurrent spectral attention module. This paper denotes the convolution as ’Conv Kernel-size Stride’, where ↑ / ↓ represent upsampling and downsampling, respectively.

As shown in Figure 3, the RSACN consists of encoder $G_{a}$, decoder $G_{s}$, hyperencoder $H_{a}$, hyperdecoder $H_{s}$, quantizer *Q*, and an entropy model. First, $G_{a}$ encodes $X_{\beta }$ into a latent representation *y*. Then, $H_{a}$ captures the spatial dependencies within *y* and represents them as *z*. *Q* ensures that stochastic gradient descent remains differentiable throughout the optimization process while quantizing *y* and *z* into discrete values $\hat{y}$ and $\hat{z}$. Here, $\hat{z}$ serves as prior information for *y* and stores the statistical information $\mu ,\hat{\sigma }$ required for arithmetic coding. Then, $\mu ,\hat{\sigma }$ are decoded through $H_{s}$ to assist the entropy model in probability modeling during encoding. Meanwhile, $\hat{y}$ is input into $G_{s}$ to reconstruct the band clusters $\hat{X_{\beta }}$. The entire compression process is formulated in Equations (5)–(7):

(5) $$y=G_{a}\left(X_{\beta },\varphi_{g}\right),z=H_{a}\left(y,\varphi_{h}\right)$$

(6) $$\hat{y}=Q\left(y\right),\hat{z}=Q\left(z\right)$$

(7) $$\mu ,\hat{\sigma }=H_{s}\left(\hat{z},\theta_{h}\right),\hat{X_{\beta }}=G_{s}\left(\hat{y},\theta_{g}\right)$$

where $\varphi_{g}$ and $\varphi_{h}$ are the optimized parameters of $G_{a}$ and $H_{a}$, and $\theta_{g}$ and $\theta_{h}$ represent the parameters learned by $G_{s}$ and $H_{s}$. During training, we replace quantization with additive uniform noise to optimize ARM-Net using stochastic gradient descent since rounding to the nearest integer produces zero gradients almost everywhere [11]. $\hat{y}$ can be modeled using a mean-scale Gaussian distribution. Non-parametric, fully factorized density models are trained on the auxiliary information $\hat{z}$.

**Figure 4:**
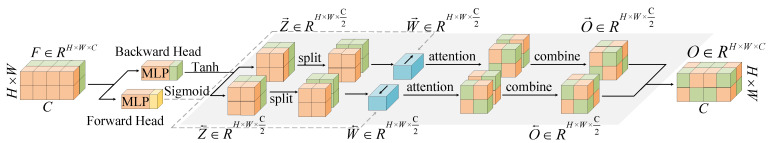
Illustration of the multi-head recurrent spectral attention block. MLP stands for two-layer linear projections with Tanh activation. C and HW denote the spectral and spatial dimensions, respectively.

Although each band in an HSI may appear visually independent, each reflects different spectral information from the same scene. Pixels at the same spatial location across different bands often share similar spectral features, which are inherently linked to the material properties of the objects. This phenomenon indicates that, despite the rich spectral data in HSIs, the bands are not isolated from one another but are intrinsically correlated. This further underscores the significant spectral correlation inherent in HSIs. Similar to non-local spatial correlations, many schemes use inter-spectral correlation for long-range dependency extraction via methods such as non-local mean (NLM) [33]. However, unlike spatially similar pixels, pixels in the spectral domain have different value ranges, which makes NLM unsuitable, as its averaging operation may disrupt the spectral relationships of each pixel across the spectrum. To address this problem, the MHRSA block [34] is inserted into the codec to dynamically compute the weights of average pixels across the spectrum for each band. Each band is assigned a distinct weight to aggregate information from other bands so that spectral dependencies are preserved. A diagram of the MHRSA block is shown in Figure 4.

It first employs two Multi-Layer Perceptrons (MLPs), followed by the application of two distinct activation functions to transform the input features into candidate features *Z* and subsequently merge the weights *W*. Specifically, we adopt tanh for generating candidate features due to its symmetric range (−1,1), which can better capture both positive and negative correlations. We use sigmoid for more interpretable and stable re-scaling of feature amplitudes.

(8) $$Z=tanh(MLP_{1}\left(F\right))$$

(9) $$W=sigmoid(\left(MLP_{2}\left(F\right)\right)$$

(10) $$MLP\left(X\right)=W_{1}*\left(tanh\left(W_{2}*X\right)\right)$$

where $W_{1},W_{2}\in R^{C\times C}$.

These processes can be equivalently viewed as the query, key, and value projections in self-attention [35]. The key difference is that the attention weights are computed directly, rather than deriving the attention map through the covariance of the key and query. Another distinction is that we perform the attention operation through a recurrent fusion step, which requires linear memory and time complexity. Specifically, the recurrent merging step for spectral mixing is performed through the accumulation of the candidate feature *Z* for each band based on the merging weight *W* as follows:

(11) $$O_{i}=\left(1-W_{i}\right)⊙Z_{i}+W_{i}⊙O_{i-1}$$

where $O_{i}$, $Z_{i}$, and $W_{i}$ are the output features, candidate features, and merging weights of the *i*-th band, respectively. It can be observed that this merging step fuses features from all previous bands $Z_{i}$, where i < j for the *j*-th band. Therefore, it links inter-spectral features and can leverage information from cleaner bands to reduce spectral redundancy.

### 2.4. Multi-Scale Spatial-Spectral Attention Reconstruction Network (MSSARN)

HSIs contain abundant spatial and spectral features. Existing transformer-based models often focus only on spatial or spectral information, which leads to information loss. To address this issue, this paper proposes a transformer-based multi-scale feature extraction module called the Multi-Scale Spatial-Spectral Attention (MSSA) module, which is embedded into the reconstruction network. MSSA is designed to extract both spatial and spectral features simultaneously, which improves reconstruction performance.

**Figure 5:**
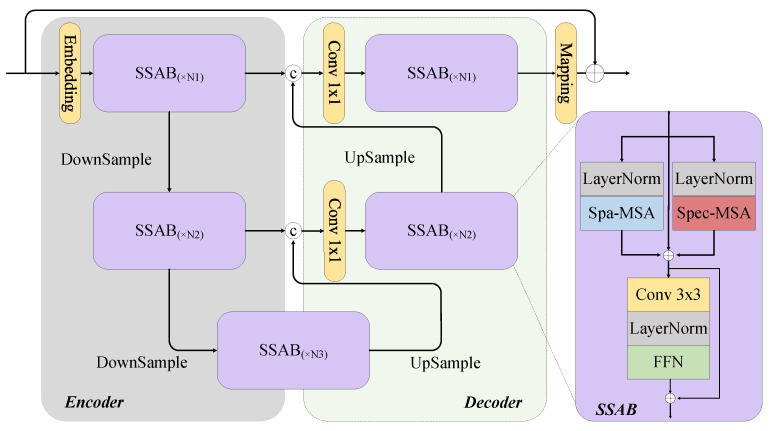
MSSA module and SSAB module ($N_{1}$, $N_{2}$, and $N_{3}$ are all set to 3).

**Figure 6:**
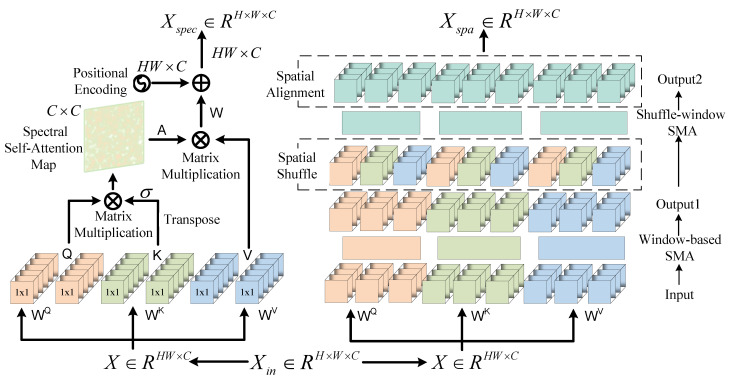
Structures of Spec-MSA and Spa-MSA.

As illustrated in Figure 1, the MSSARN consists of three cascaded MSSA modules. Direct use of transformer-based approaches may result in computational error accumulation due to the attention mechanism’s emphasis on long-range similarity [36]. To mitigate this, we adopt a U-shaped transformer architecture for fine-grained reconstruction. Additionally, a two-dimensional attention mechanism is employed to capture long-range dependencies between spectral bands. As depicted in Figure 5, MSSA employs U-Net as the backbone for the top-down extraction of effective features. The convolutional layers before and after MSSA, the embedding block, and the mapping block are single convolutional 3 × 3 layers. During the encoder stage, $N_{1}+N_{2}$ SSABs are used to extract hierarchical abstract features. Simultaneously, convolutional 4 × 4 downsampling operations reduce spatial resolution and increase channel depth. The decoder follows a symmetric structure that utilizes 2 × 2 deconvolutions for upsampling and $N_{1}+N_{2}$ SSABs to progressively integrate features. The bottleneck layer comprises $N_{3}$ SSABs. To preserve information lost during downsampling, skip connections are introduced between the encoder and decoder. A 1 × 1 convolution is applied to fuse spatial and spectral features. The right side of Figure 5 illustrates that each SSAB consists of a Feed-Forward Network (FFN), Spec-MSA [37], Spa-MSA [38], and LayerNorm. The convolutional layer is a single convolutional 3 × 3 layer. The FFN follows the parameter settings outlined in [37]. Additionally, window-based transformers often encounter the ’grid issue’ when processing high-resolution images. To alleviate this, we introduce residual connections between window-based MSA and shuffle-window MSA to enhance feature interaction. The convolutional kernel size is set to be matched with the window size to ensure alignment in feature extraction. As illustrated in Figure 6, the spatial-spectral attention mechanism comprises parallel Spa-MSA and Spec-MSA modules that compute spatial and spectral multi-head self-attention, respectively. The two attention modules operate in parallel to provide dual features that enhance cross-dimensional interactions.

**Figure 7:**
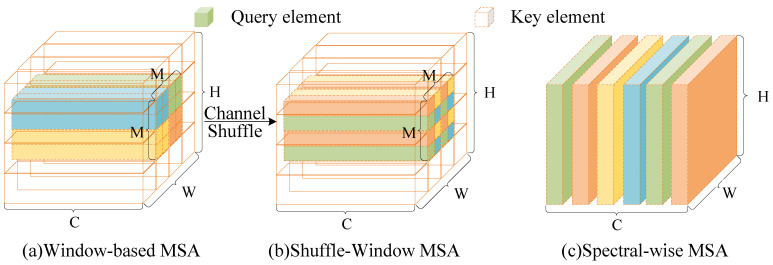
Diagram of different MSAs. The green-colored box represents the query element, and the dashed box denotes the key element. (**a**) W-MSA calculates self-attention within position-specific windows. (**b**) SW-MSA processes data from different windows after W-MSA through spatial shuffling and alignment, introducing global cross-window interaction. (**c**) Spec-MSA treats each spectral channel as a token and calculates the self-attention along the spectral dimension.

As illustrated on the left side of Figure 6 and Figure 7c, Spec-MSA treats each spectrum as a token and thus focuses on more non-local spectral self-similarities.

Spec-MSA computes self-attention for $head_{j}$:

(12) $$A_{j}=\mathrm{softmax}\left(\sigma_{j}K_{j}^{T}Q_{j}\right),\;head_{j}=V_{j}A_{j}$$

where $K_{j}^{T}$ is the transpose of $K_{j}$.

Due to the significant variation in spectral density across wavelengths, a learnable parameter $\sigma_{j}$ is used to adapt the self-attention $A_{j}$ by reweighting the matrix multiplication $K_{j}^{T}$$Q_{j}$ within $head_{j}$. The softmax function is applied to normalize the attention weights across all spectral tokens, ensuring that the computed attention scores form a probability distribution and highlight key spectral correlations. Subsequently, the outputs of *N* heads are concatenated and passed through a linear projection, followed by the incorporation of positional embeddings:

(13) $$Spec-MSA\left(X\right)=\left(\mathrm{Concat}\left(head_{j}\right)\right)W+f_{p}\left(V\right)$$

where $W\in R^{C\times C}$ represents the learnable parameters and $f_{p}\left(\cdot \right)$ is the function to generate positional embeddings.

It consists of two depth-wise 3 × 3 convolutional layers, a Gaussian error linear unit activation function, and reshaping operations. The HSI is ordered along the spectral dimension by wavelength. Therefore, these embeddings are utilized to encode the positional information of different spectral channels. Finally, the result of Equation (13) is reshaped to obtain the output feature map $X_{spec}\in R^{H\times W\times C}$.

Spa-MSA consists of a window-based MSA (W-MSA) and a shuffle-window MSA (SW-MSA), which are designed to facilitate long-range cross-window interactions. The right side of Figure 6 and Figure 7a,b depicts W-MSA and SW-MSA, with their primary difference being the spatial shuffle mechanism. In brief, for a W-MSA with window size M and N tokens as input, the output is reshaped to (M, N/M), transposed, and flattened to serve as input for the next layer. This combines tokens from different windows to establish long-range connections. Subsequently, the spatial dimensions are reshaped to (N/M, M) through spatial alignment operations with relative positional offsets, followed by transposition and flattening to restore their original configurations.

## 3. Results

### 3.1. Experimental Configurations

**Figure 8:**
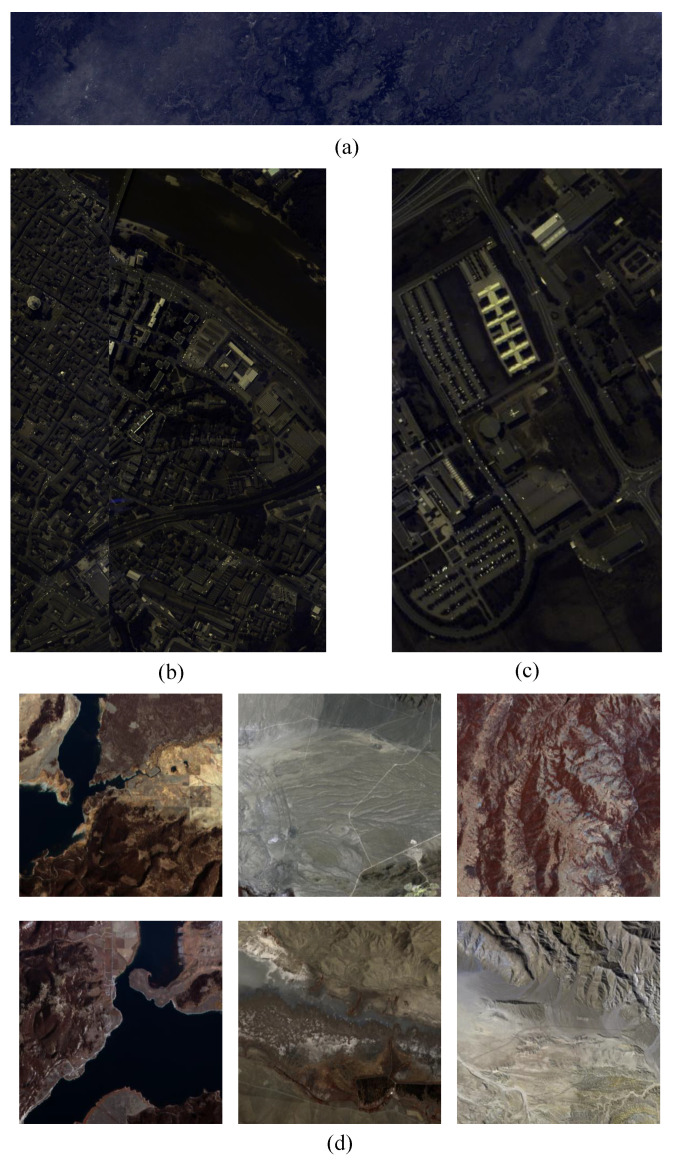
(**a**) Botswana dataset. (**b**) Pavia Center dataset. (**c**) Pavia University dataset. (**d**) Examples of other HSIs collected by the AVIRIS sensor.

To thoroughly assess the proposed HSI compression architecture and the ARM-Net designed within this framework, this paper trained ARM-Net on a large, high-quality mixed HSI dataset, allowing us to test our model on new HSIs without the need for retraining. The dataset integrates HSI data from several well-known HSI datasets, including Botswana, KSC, Pavia Center, Pavia University, Salinas, and Houston, as well as hyperspectral data collected by the AVIRIS sensor [39]. The spatial resolution of the dataset ranges from 512 × 217 to 2384 × 601. For efficient processing, the dataset was first divided into non-overlapping patches of 128 × 128, and then 30-channel random overlapping sub-patches were generated along the spectral dimension. Data augmentation techniques, such as horizontal flipping, vertical flipping, and rotation, were applied to enhance the diversity and robustness of the data. In the end, 26,380 patches were randomly divided into training, validation, and test sets in a ratio of 8:1:1. Figure 8 presents a selection of images from the dataset to highlight the diversity and richness of the hyperspectral data. Various scenes within the integrated hyperspectral dataset are illustrated in these images, showcasing different spectral characteristics and visual details. To effectively visualize different regions, the input image was transformed into a pseudo-color image, allowing for a clearer differentiation of areas to meet human perceptual requirements.

### 3.2. Training Details

To ensure efficient compression and reconstruction, ARM-Net was trained in two stages. First, the training set was normalized using divergence normalization, and then RD loss was applied to train the compression network, as shown in Equations (14)–(16):

(14) $$Loss_{com}=R\left(\hat{y}\right)+R\left(\hat{z}\right)+\lambda *D\left(X_{\beta },\hat{X_{\beta }}\right)$$

(15) $$R\left(\hat{y}\right)=E\left[-\log_{2}\left(N\left(\mu ,\sigma^{2}\right)*U\left(-\frac{1}{2},\frac{1}{2}\right)\left(\hat{y}\right)\right)\right]$$

(16) $$R\left(\hat{z}\right)=E\left[-\log_{2}\left(P_{\hat{z}|\psi }\left(\hat{z}|\psi \right)*U\left(-\frac{1}{2},\frac{1}{2}\right)\left(\hat{z}\right)\right)\right]$$

where ψ represents the parameters of the Gaussian distribution, *R* represents the bit rate, and *D* is the distortion between the original and reconstructed images. The Mean Squared Error (MSE) was used as the metric to measure *D*. λ is the Lagrange multiplier, a hyperparameter used to balance the bit rate and reconstruction distortion. After the RSACN converged, these parameters were frozen, and the MSSARN was trained using reconstruction distortion, as shown in Equation (17):

(17) $$Loss_{rec}=MSE\left(X_{\mathrm{in}},{\hat{X}}_{\mathrm{in}}\right)$$

The entire model was trained for 1800 epochs with a batch size of 20. The network was optimized using the Adam optimizer with an initial learning rate of 0.0001. When there were no significant changes in the RD loss, the learning rate was reduced to 0.00005. The entire network was accelerated and optimized using an NVIDIA 3090 GPU. All codecs were run on the same CPU (i7-12700H @ 2.3 GHz) during inference.

### 3.3. Evaluation Strategies

In this paper, all deep learning-based methods were evaluated using the PSNR metric. The compression ratio of HSIs was measured by the number of bits per pixel per band (bpppb), and three commonly used metrics were employed to assess the distortion introduced during the compression process from different perspectives: PSNR [24], MS-SSIM [24], and SAM [22], with the following calculation formulas:

(18) $$PSNR\left(X,\hat{X}\right)=\frac{1}{C}\sum_{i=1}^{C}10\log_{10}\left(\frac{\max^{2}\left(X_{i}\right)}{{\mathrm{MSE}}_{i}}\right)$$

where $\mathrm{MSE}\left(X,\hat{X}\right)=\left(\frac{1}{H\times W\times C}\right){∥X-\hat{X}∥}_{F}^{2}$ and $\max^{2}\left(\cdot \right)$ denotes the square of the maximum pixel in the *i*-th band.

(19) $$SAM\left(X,\hat{X}\right)=\frac{1}{H\times W}\times \sum_{j=1}^{H\times W}\cos^{-1}\left(\frac{X_{j}\cdot \hat{X_{j}}}{∥X_{j}∥∥\hat{X_{j}}∥}\right)$$

where · represents the inner product, ∥·∥ denotes the $L_{2}$ norm, and $X_{j}$ and ${\hat{X}}_{j}$ denote the *j*-th pixel of the original and reconstructed HSIs, respectively.

MS-SSIM [11] is expressed in decibels as $-10\log_{10}\left(1-\mathrm{MS}-\mathrm{SSIM}\right)$. Higher PSNR and MS-SSIM values indicate better spatial fidelity, while a lower SAM value signifies better spectral fidelity [37].

### 3.4. Comparative Results

#### 3.4.1. Rate-Distortion Performance

This study compared nine compression methods, including three hyperspectral image compression methods: ARM-Net (ours), FHNeRF (2024) [24], and Verdú (2024) [26]; three RGB image compression methods: CHENG (2020) [17], Pan (2023) [40], and Hyperprior (2017) [11]; one traditional hyperspectral imaging method: PCA [9]; and two traditional algorithms: BPG and JPEG2000. The traditional compression methods utilize publicly available software or code. Specifically, JPEG2000 was implemented using the OpenJPEG library, BPG is based on an open-source C language library, and PCA was performed by combining PCA with JPEG2000. Other methods followed the same setup as described in the original studies. Table 1 presents all the methods mentioned in the comparative experiments along with their key characteristics.

**Figure 9:**
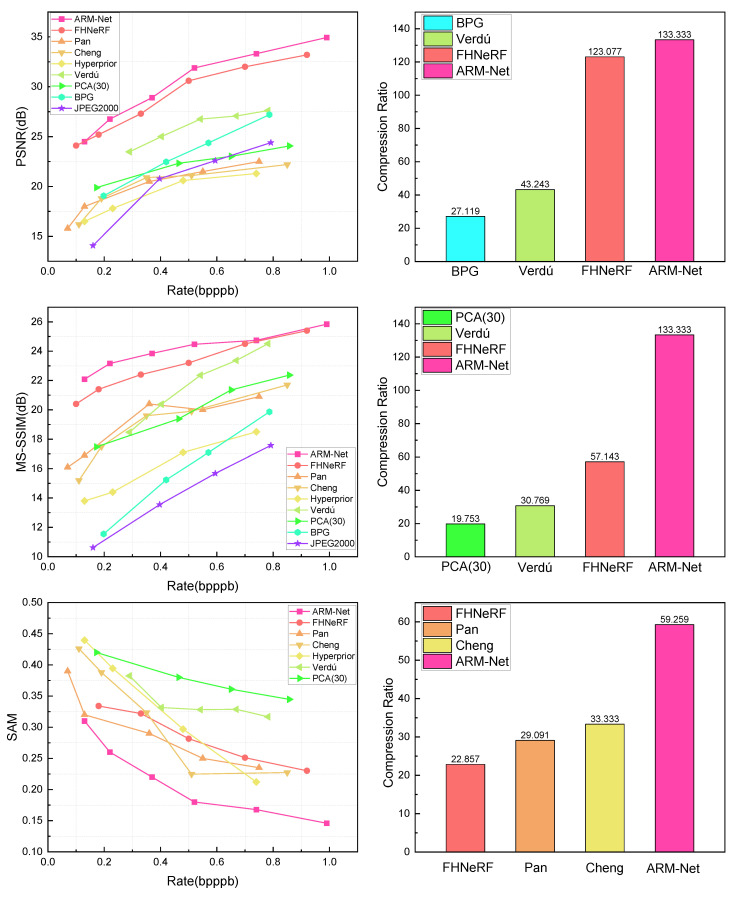
RD curves in terms of PSNR, MS-SSIM, and SAM aggregated over the mixed dataset. The plots on the **left**, from top to bottom, illustrate bpppb vs. PSNR, bpppb vs. MS-SSIM, and bpppb vs. SAM, respectively. The plots on the **right** present the compression ratios. Our proposed ARM-Net (bright pink line) achieves better RD performance than other approaches.

Figure 9 presents the RD curves and compression ratios of various compression methods on the mixed dataset. Not surprisingly, our ARM-Net was highly competitive and outperformed most existing methods. In the context of high compression ratios, the reconstruction quality of deep learning approaches [11,17,24,26,40] is primarily contingent upon the network’s feature inference capabilities. ARM-Net and [24,26] benefited from the incorporation of sophisticated feature extraction modules and feature-domain information flow channels, which together enabled them to achieve reconstruction quality superior to that of traditional algorithms. ARM-Net employs a cyclic spectral information fusion methodology, integrating spatial texture and spectral correlation. It outperformed the other methods at both low and high compression ratios. Specifically, ARM-Net achieved a 1.09 dB improvement in PSNR over the SOTA methods. At a bpppb of 0.58, ARM-Net’s PSNR was approximately 1.5 dB higher than that of FHNerF [24]. Furthermore, ARM-Net exhibited minimal fluctuations across different bit rates, indicating its ability to maintain stable reconstruction quality. Additionally, as the compression ratio decreased, the amount of information in the observed values increased. Existing methods, which typically rely on complex feature processing and optimization steps, iteratively refine the reconstructed image. In contrast, ARM-Net leverages an innovative network architecture to perform feature extraction and optimization directly on the sampled bands, thereby avoiding the complexity of iterative steps and enabling more efficient image reconstruction.

#### 3.4.2. Comparison of Visualization Results

**Figure 10:**
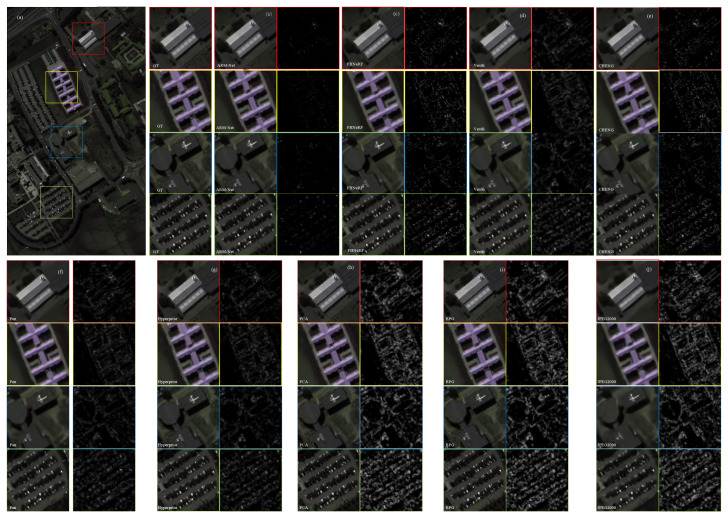
Visual comparison of example blocks from the Pavia University dataset at high bit rates. From (**a**–**j**): Ground Truth (GT), ARM-Net (bpppb: 0.74; PSNR: 33.32 dB; MS-SSIM: 24.74 dB; SAM: 0.17), FHNeRF (bpppb: 0.70; PSNR: 31.99 dB; MS-SSIM: 24.5 dB; SAM: 0.25), Verdú (bpppb: 0.78; PSNR: 27.62 dB; MS-SSIM: 24.51 dB; SAM: 0.33), CHENG (bpppb: 0.85; PSNR: 22.29 dB; MS-SSIM: 21.74 dB; SAM: 0.23), Pan (bpppb: 0.75; PSNR: 22.51 dB; MS-SSIM: 20.90 dB; SAM: 0.22), Hyperprior (bpppb: 0.74; PSNR: 21.36 dB; MS-SSIM: 18.58 dB; SAM: 0.212), PCA (bpppb: 0.86; PSNR: 24.07 dB; MS-SSIM: 22.37 dB; SAM: 0.35), BPG (bpppb: 0.79; PSNR: 27.20 dB; MS-SSIM: 19.86 dB; SAM: 0.34), and JPEG2000 (bpppb: 0.7918; PSNR: 24.4 dB; MS-SSIM: 17.58 dB; SAM: 0.06).

Figure 10 shows a visual comparison of a test patch from Pavia University containing numerous small buildings. The quantitative results for PSNR, MS-SSIM, and SAM are also displayed below the corresponding visualizations. Notably, the bpppb values presented in the visual comparison exhibit slight fluctuations. This variation arose because the reported values were based on actual measurements rather than data points extracted from the curves in the figure. The results indicate that the results of both classical codecs (JPEG2000, PCA, and BPG) and deep learning-based methods suffered from varying degrees of blur. The reconstructed image of ARM-Net (b) displays excellent texture details and edge definition. Although FHNerF (c) performed well at high bit rates, it exhibited a slightly lower PSNR, resulting in some loss of detail. Verdú (d) demonstrated a substantial degradation in reconstruction quality, accompanied by noticeable texture blurring. Other methods, such as CHENG (e), Pan (f), and Hyperprior (g), fell short in terms of texture details and contrast. In general, ARM-Net demonstrated superior visual perception compared to the other compression methods.

**Figure 11:**
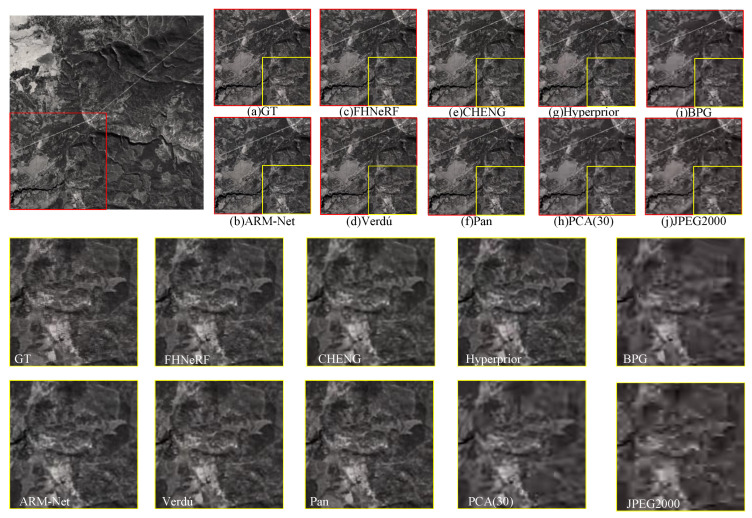
Visual comparison of example data blocks from the ARIVIRS sensor acquisition dataset at low bit rates. (**a**) Original. (**b**) ARM-Net (bpppb: 0.22; PSNR: 26.76 dB; MS-SSIM: 23.16 dB; SAM: 0.26). (**c**) FHNerF (bpppb: 0.18; PSNR: 25.20 dB; MS-SSIM:21.40 dB; SAM: 0.33). (**d**) Verdú (bpppb: 0.28; PSNR: 23.48 dB; MS-SSIM: 18.47 dB; SAM: 0.34). (**e**) CHENG (bpppb: 0.19; PSNR: 18.85 dB; MS-SSIM: 17.56 dB; SAM: 0.38). (**f**) Pan (bpppb: 0.13; PSNR: 18.10 dB; MS-SSIM: 16.90 dB; SAM: 0.32). (**g**) Hyperprior (bpppb: 0.23; PSNR: 17.81 dB; MS-SSIM: 14.41 dB; SAM: 0.39). (**h**) PCA (bpppb: 0.17; PSNR: 19.88 dB; MS-SSIM: 17.47 dB; SAM: 0.42). (**i**) BPG (bpppb: 0.20; PSNR: 19.07 dB; MS-SSIM: 11.55 dB; SAM: 0.40). (**j**) JPEG2000 (bpppb:0.16; PSNR: 14.08dB; MS-SSIM: 10.62 dB; SAM: 0.12).

This study also conducted experiments on HSI data collected by AVIRIS, which cover objects of different scales and types, to further validate the effectiveness of ARM-Net. Figure 11 presents a visual comparison of a sample patch from the AVIRIS dataset at low bit rates. The dataset predominantly contains complex mountain textures, characterized by highly irregular structures and rich spatial details, which presents a significant challenge for compression algorithms. Traditional methods of dealing with such complex textures at low bit rates exhibited noticeable shortcomings. Severe detail loss was observed in CHENG (e) during reconstruction. The mountain textures appeared blurred, and the contrast was reduced, causing the structure to look flattened. Similarly, Hyperprior (g) failed to restore the intricate mountain details, as obvious noise and distorted textures appeared in the reconstructed image. Furthermore, PCA (h) did not adequately preserve the mountain details during compression, resulting in blurred image edges. In contrast, ARM-Net effectively mitigated structural distortions during compression by combining spatial and spectral information via the SSAB, which operates bidirectionally. ARM-Net successfully preserved the detailed texture of the mountain ranges and avoided the structural distortions that occurred with other methods. This resulted in better reconstruction quality, further confirming its advantages in complex natural scenes.

#### 3.4.3. Model Complexity Analysis

Table 2 lists the parameters, FLOPs, and inference times for the six learning-based comparison methods. Compared to other methods, ARM-Net maintained a good parameter count and achieved a significant reduction in computational complexity. The number of parameters was slightly higher than that of Hyperprior and Verdú due to the three-segment structure of ARM-Net. Similarly, ARM-Net, downscaled with an adaptive band selector, had significantly fewer FLOPs than other end-to-end compression networks. FHNeRF achieved a lower number of parameters and computational complexity via the representation of pixel coordinates. While FHNeRF excelled across all metrics, it required a significant amount of training and performed slightly worse than our method in terms of PSNR and MS-SSIM. This indicates that ARM-Net had a moderate inference time. Additionally, since the reconstruction network needs to capture spatial and spectral features, its decoding phase took longer than that of the end-to-end compression network.

### 3.5. Ablation Experiments

#### 3.5.1. Ablation Experiments on Band Selection

Notably, the upper limit of the reconstruction network’s performance is determined by the sampled band clusters. Specifically, if the image information provided to the reconstruction network is incomplete, it becomes challenging for the network to adapt to the full band information. Our band correlation algorithm evaluates and samples groups of bands with large differences to ensure the information richness of the input image, which leads to an improvement in reconstruction quality. However, since band data are not uniformly distributed, certain bands may contain crucial information for the reconstruction task and equidistant sampling might miss these key bands, causing a loss of important information. This deficiency is particularly evident in the MSE loss curve and the PSNR metric. Under identical conditions, Table 3 shows the PSNR performance of the equidistantly sampled band clusters [1, 7, 13, 19, 25, 30] and the band clusters [1, 2, 20, 25, 29, 30] selected by the adaptive band selection algorithm at the same compression rate. It can be seen that the PSNR of the band clusters sampled by the adaptive band selection algorithm was about 2–3 dB higher than that of the equidistant sampling method. This indicates that the adaptive band selection algorithm contributed to the improvement of the reconstruction network’s performance.

We also tested the hyperparameter β with different values to observe PSNR metrics under the same bpppb. Table 4 indicates that when β=2, the band clusters did not adequately represent the patch, as evidenced by the lower PSNR. On the other hand, β=4 did not result in a higher PSNR gain but increased the network’s parameter count.

**Figure 12:**
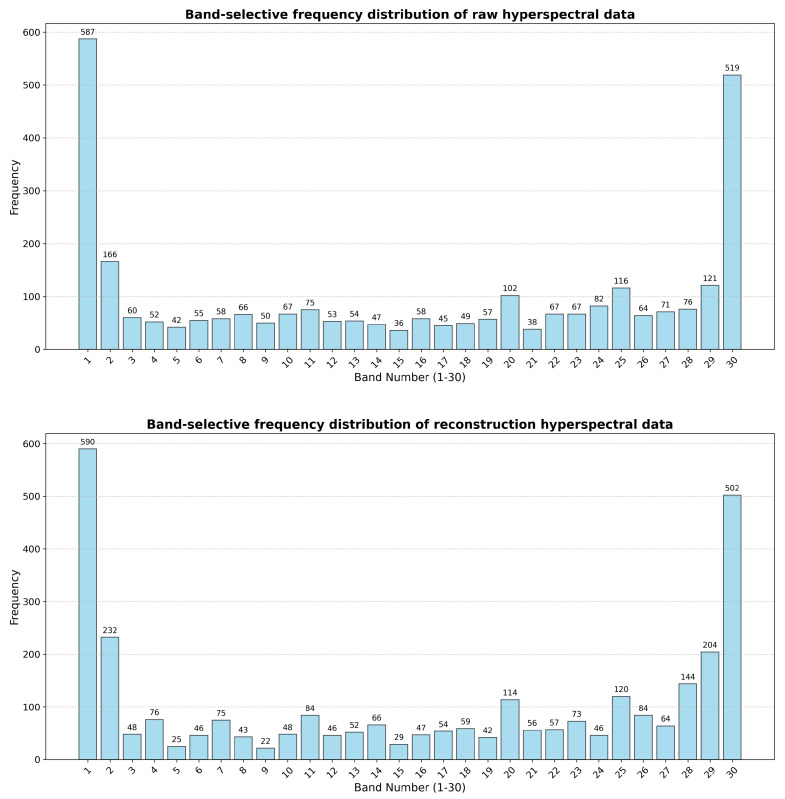
Band-selective frequency distribution comparison of original and reconstructed hyperspectral data. The horizontal axis (1–30) represents the 30 bands of the patch, while the vertical axis shows the frequency with which each band was selected by the ABS.

To further validate the performance of the ABS, this experiment first applied the ABS to select bands from 500 randomly selected HSI patches. These 500 patches were then input into the trained compression model for compression and reconstruction. The ABS was applied again to the reconstructed HSI to observe differences between the band selection results of the reconstructed HSI and the raw HSI before compression. A high degree of overlap between the reconstructed data and the band selection results of the original inputs would indicate a strong ability of the model to identify and preserve key spectral information. Conversely, significant deviations may highlight potential limitations in the model’s ability to extract meaningful features during the reconstruction process. Figure 12 shows that the reconstructed band correlations are nearly identical to the original input data and consistent with the results of adaptive band selection. This confirms that the subsequent compression and reconstruction processes are meaningful.

#### 3.5.2. Ablation Experiments on the Attention Module

**Figure 13:**
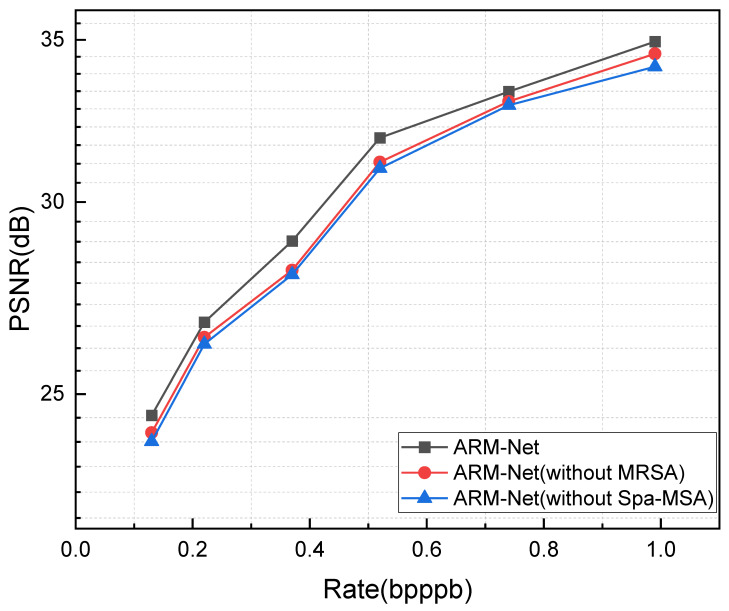
Comparison of MRSA and Spa-MSA.

To test the impact of Spa-MSA and MRSA on overall compression performance, the following configurations were evaluated. First, a baseline model without the spatial attention module was used to assess its performance in terms of compression effectiveness and distortion metrics. Next, a model without MRSA was evaluated to assess performance based solely on the spatial attention module. Finally, both modules were applied simultaneously to the model to comprehensively assess their combined effects. The results shown in Figure 13 demonstrate removing both MRSA and Spa-MSA led to a decrease in PSNR. Specifically, when MRSA was removed, PSNR decreased across various bit rates, although the overall decline remained relatively small. In contrast, the removal of Spa-MSA resulted in a more significant drop in PSNR, particularly at lower bit rates. Compared to the complete ARM-Net model, this performance degradation confirms that both MRSA and Spa-MSA are essential for refining compression quality.

#### 3.5.3. Ablation Experiments on the Framework

**Figure 14:**
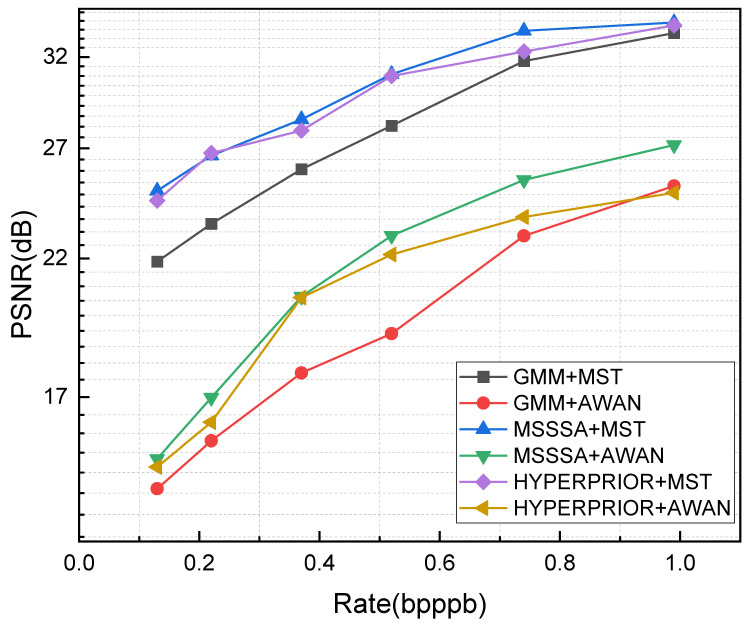
PSNR values achieved by different network combinations in the ablation experiments.

This section presents a series of ablation experiments on the proposed three-stage framework to evaluate the impact of different combinations of compression and reconstruction networks on overall compression performance. As shown in Figure 14, three learned compression methods—Hyperprior [11], MSSSA [20], and GMM [17]—and two SOTA SR networks—MST [37] and AWAN [41]—were embedded into the proposed framework, and their combined PSNR performance was measured. Table 5 provides detailed information on the network parameters, FLOPs, and average inference time for each combination. The average inference time represents the mean duration for the network to complete compression during testing, apart from the arithmetic encoding and decoding processes. As can be seen in Figure 14 and Table 5, the MSSSA+MST achieved the highest PSNR gain but also significantly increased the computational load, approximately five times that of Hyperprior+MST. GMM was outperformed by the other two compression networks within the framework. Furthermore, MST surpassed AWAN in reconstruction capability. From a combined perspective of performance and computational complexity, Hyperprior+MST, as a baseline for the framework, is by far the best choice.

## 4. Discussion

ARM-Net employs the proposed three-stage composite framework, with the compression architecture incorporating the MHRSA module and the reconstruction architecture integrating a multi-scale spatial-spectral attention mechanism. The objective is to adaptively learn robust spectral and spatial representations through attention mechanisms, guided by the inherent structure of hyperspectral data. As shown in Figure 9, Figure 10 and Figure 11, ARM-Net significantly outperforms existing compression methods, with notable improvements in PSNR and MS-SSIM, as well as a reduction in SAM. The proposed ARM-Net effectively distinguishes useful spectral and spatial features, thereby enhancing compression performance. Furthermore, ARM-Net’s spatial-spectral attention mechanism allows for detailed feature extraction across both spatial and spectral dimensions, which improves reconstruction accuracy and ensures high-quality output. Figure 14 further demonstrates that, even with a relatively simple compression network, the three-stage compression framework leads to significant performance gains. In general, ARM-Net has two significant advantages. On the one hand, the multi-scale attention mechanism selectively provides structured representations and allows for more precise pixel connections during reconstruction without introducing excessive bit rates. On the other hand, the spatial-spectral attention mechanism optimizes the compression model, which ensures that the model learns in a more structured direction and effectively balances compression efficiency with reconstruction quality.

## 5. Conclusions

This study introduces ARM-Net, a novel three-stage compression network designed to address two major HSI compression challenges: high computational complexity and the limited efficiency of spectral redundancy elimination. By leveraging the novel three-stage hybrid architecture, ARM-Net integrates an ABS, RSACN, and MSSARN to significantly enhance compression performance. In the reconstruction stage, the integration of the SSAB enables detailed feature extraction across both spatial and spectral dimensions, significantly enhancing reconstruction accuracy and ensuring that the final output retains essential visual and spectral characteristics. Additionally, the MHRSA module dynamically adjusts the weighting of each spectral band to highlight key features and reduce redundancy. Through extensive evaluations of benchmark datasets, ARM-Net demonstrates superior compression performance and achieves high detail retention and minimal distortion compared to existing methods. The qualitative results demonstrate that ARM-Net achieves high-quality compression while effectively preserving both the visual clarity and spectral consistency of reconstructed images. However, the performance of ARM-Net is still constrained by the band selection algorithm and the reconstruction network. In particular, datasets with extremely wide spectral ranges, substantial intra-band variability, or large spatial dimensions can exceed the current capacity of the model to accurately capture and reconstruct all critical features, especially when the training data do not comprehensively cover all variations. At very aggressive compression ratios, important spectral or spatial information may be lost, further exacerbating reconstruction errors. Despite these limitations, ARM-Net holds great promise for real-world HSI applications, such as in agriculture and industry. In agricultural scenarios, precise yet efficient hyperspectral data analysis can enable early detection of crop stress or disease, soil property assessment, and optimized resource management. In industrial contexts, accurate spectral reconstruction can facilitate quality inspection, material identification, and contamination detection while reducing data storage and transmission overhead. By providing both high compression ratios and fine spectral preservation, ARM-Net can support faster data-driven decisions. Future work should therefore concentrate on exploring optimized combinations of band sampling strategies and reconstruction networks. This includes refining the band selection approach to better accommodate large or highly variable datasets, as well as enhancing reconstruction models to manage more extreme compression levels without sacrificing essential spectral or spatial details. By addressing these limitations, ARM-Net has the potential to become even more robust and versatile in a broader range of hyperspectral compression scenarios, ultimately benefiting practical HSI applications in agriculture, industry, and beyond.

## Figures and Tables

**Table 1:** Summary of the nine compression methods compared in this study.

Method	Advantages	Disadvantages	Applicability	Limitations
ARM-Net (ours)	Spatial and spectral feature fusion	Framework dependency issues	General hyperspectral images	Slow decoding speed
FHNeRF (2024)	Implicit transform coding	Limited generalizability	General hyperspectral images	Training relies on specific images
Verdú (2024)	Channel clustering reduces complexity	Spectral channel dependence	General hyperspectral images	Limited by embedded architecture
CHENG (2020)	Accurate modeling of discrete Gaussian mixture models	High computational complexity	General still images	Weak spectral information representation
Pan (2023)	Focuses on content and texture branches	High computational complexity	General still images	May introduce artifacts
Hyperprior (2017)	Accurate modeling of hyperprior entropy model	Insufficient adaptability	General still images	Weak spectral information representation
PCA	Reduces feature dimensionality	Sensitive to data accuracy	Pre-compression of small-sized and high-relevance images	Complex decompression
BPG	High dynamic range	Low codec performance	High-quality, low-bandwidth transmission	Poor compatibility
JPEG2000	Transparent progressive	Low bit-rate blur	Medical/Satellite images	Limited adaptability to complex scenarios

**Table 2:** Model parameters, FLOPs, and inference times (including enc-time and dec-time) for the proposed method and comparison methods.

Method	Parameters (M)	FLOPs (G)	Enc-Times (s)	Dec-Times (s)
ARM-Net	9.3	7.9	0.16	0.29
FHNeRF	0.004785	1.7	0.11	0.14
Pan	21.0	55.6	0.42	0.40
Cheng	18.0	61.1	0.40	1.50
Hyperprior	7.1	28.7	0.12	0.15
Verdú	7.1	28.7	0.13	0.16

**Table 3:** Effect of different band selection methods on the PSNR of ARM-Net.

Compression Ratio	1.0/16	0.8/16
PSNR with adaptive band selection algorithm	34.14 dB	33.11 dB
PSNR for equally spaced samples	32.07 dB	31.66 dB

**Table 4:** Impact of hyperparameter β on PSNR performance of ARM-Net at different bpppb values.

β	2	3	4
PSNR (bpppb = 1.0)	28.14	34.14	34.21
PSNR (bpppb = 0.8)	26.11	33.11	33.06

**Table 5:** Parameters, FLOPs, and inference times of the ablation methods.

Methods	Parameters	FLOPs	Times
Hyperprior+MST	8.6 M	8.0 G	16.8 ms
MSSSA+MST	38.7 M	45.5 G	30.8 ms
Cheng+MST	11.2 M	11.5 G	21.6 ms
Hyperprior+AWAN	7.5 M	9.9 G	17.9 ms
MSSSA+AWAN	37.5 M	47.2 G	34.1 ms
Cheng+AWAN	10.1 M	13.6 G	22.6 ms

## Data Availability

Data are contained within the article.
